# Phenomic and genomic prediction of yield on multiple locations in winter wheat

**DOI:** 10.3389/fgene.2023.1164935

**Published:** 2023-05-09

**Authors:** Robert Jackson, Jaap B. Buntjer, Alison R. Bentley, Jacob Lage, Ed Byrne, Chris Burt, Peter Jack, Simon Berry, Edward Flatman, Bruno Poupard, Stephen Smith, Charlotte Hayes, Tobias Barber, Bethany Love, R. Chris Gaynor, Gregor Gorjanc, Phil Howell, Ian J. Mackay, John M. Hickey, Eric S. Ober

**Affiliations:** ^1^ The John Bingham Laboratory, NIAB, Cambridge, United Kingdom; ^2^ The Roslin Institute and Royal (Dick) School of Veterinary Studies, The University of Edinburgh, Scotland, United Kingdom; ^3^ KWS UK Ltd, Thriplow, Royston, Cambridgeshire, United Kingdom; ^4^ RAGT UK, Ickleton, Saffron Walden, Cambridgeshire, United Kingdom; ^5^ Limagrain UK Ltd, Rothwell, Market Rasen, Lincolnshire, United Kingdom; ^6^ Elsoms Wheat Limited, Spalding, Linconshire, United Kingdom

**Keywords:** wheat, yield, genomic selection, phenomics, GxE, remote sensing, hyperspectral

## Abstract

Genomic selection has recently become an established part of breeding strategies in cereals. However, a limitation of linear genomic prediction models for complex traits such as yield is that these are unable to accommodate Genotype by Environment effects, which are commonly observed over trials on multiple locations. In this study, we investigated how this environmental variation can be captured by the collection of a large number of phenomic markers using high-throughput field phenotyping and whether it can increase GS prediction accuracy. For this purpose, 44 winter wheat (*Triticum aestivum* L.) elite populations, comprising 2,994 lines, were grown on two sites over 2 years, to approximate the size of trials in a practical breeding programme. At various growth stages, remote sensing data from multi- and hyperspectral cameras, as well as traditional ground-based visual crop assessment scores, were collected with approximately 100 different data variables collected per plot. The predictive power for grain yield was tested for the various data types, with or without genome-wide marker data sets. Models using phenomic traits alone had a greater predictive value (R^2^ = 0.39–0.47) than genomic data (approximately R^2^ = 0.1). The average improvement in predictive power by combining trait and marker data was 6%–12% over the best phenomic-only model, and performed best when data from one full location was used to predict the yield on an entire second location. The results suggest that genetic gain in breeding programmes can be increased by utilisation of large numbers of phenotypic variables using remote sensing in field trials, although at what stage of the breeding cycle phenomic selection could be most profitably applied remains to be answered.

## Introduction

The amount of data provided by genotyping technologies outweighs that provided by phenotyping. This has led to phenotyping being labelled as the current “bottleneck” in plant breeding (Furbank and Tester, 2011). In the past decade, the deployment of remote sensing technologies has resulted in a large increase in the amount of phenotyping data that can be collected from plant breeding field trials (Atkinson et al., 2018). With this advancement came the new problems of how best to utilise large volumes of phenotype data and how to combine it with genotyping outputs (Shakoor et al., 2019). In this paper we aim to show how phenomic data can be used to predict yields and how it can be combined with genomic prediction to increase the prediction accuracy of both methods.

Phenomics is the study of physiological, biochemical and morphological traits of an individual and the corresponding genetic and environmental factors (Deshmukh et al., 2014). In plant breeding terms, this is the global study of phenotypes relating to growth and development throughout the growing season. Most classic methods of collecting phenotypic data, such as visual scoring, are slow, subjective, and labour intensive. Advancements in remote sensing have provided quick and reliable methods of collecting numerous data points simultaneously, in a short period of time (Atkinson et al., 2018). In addition, soil scanning technologies now allow for the measurement of topsoil depth, soil texture and moisture content (Doolittle and Brevik, 2014). Together with traditional ground based visual scoring (e.g., growth staging) it is now possible to produce a large phenomic data resource, comparable to that produced by genomics, for each trial. As a result, remote sensing is increasingly used to systematically collect field data on a large scale at reasonable costs. The significance of this high-throughput field phenotyping for plant genetics and breeding has been reviewed by Cabrera-Bosquet et al. (2012), Araus et al. (2018), Atkinson et al. (2018) and Rebetzke et al. (2019).

Genomic selection, which allows prediction of performance based on large genome-wide marker datasets, has been applied in wheat breeding for over two decades (Meuwissen et al., 2001). In genomic selection, part of the germplasm under selection is not extensively phenotyped but its performance is predicted by a statistical model, which is based on the phenotypic performance measurements of a training population of related materials and dense genotype data of all materials (Jannink et al., 2010). By making selections on the basis of genotype without the need to phenotype the individuals accelerates the breeding cycle and saves time and resources. However, the phenotypic performance of the training population can change depending on the location or year of the trial (Mackay et al., 2015). This is because the expression of most quantitative traits such as yield are not only determined by the genotype but are also influenced by the environmental conditions at a particular location. Thus, these genotype-by-environment interactions (GxE) are an intrinsic hurdle for the efficacy of genomic prediction in crops. Consequently, a genomic prediction model that has been trained on a single location may have a very high prediction accuracy when cross-validating over environmentally similar locations but may have poor prediction on related or even the same genetic materials grown at different locations. A solution to this problem is to grow the training population in multiple locations which will, in turn, negate the benefits of genomic selection. An alternative solution is to measure environmental variation directly, and incorporate these covariates in a prediction model (Mackay et al., 2015). For example, variation in soil characteristics across a trial can be quantified by measurements of apparent soil conductivity and soil nutrient levels. In addition, environmental effects can be captured indirectly via phenotypic measurements of plants that reflect variations in plant development or the physiological status of tissues. For example, remote sensing methods can be used to derive vegetation indices based on spectral reflectance signatures of the canopy. Therefore, the combination of phenomic and genomic markers provides a dataset capable of accounting for genotype by environment interactions.

In the past decade a number of approaches have been used to directly or indirectly take into account the impact of environmental variation on yield prediction in wheat. Heslot et al. (2014) used weather data and plant growth models to improve predictions over 44 environments. Rutkoski et al. (2016) and Crain et al. (2018) demonstrated the use of a limited number of remote sensing traits as covariates for yield prediction. Ovenden et al. (2018) used factor analysis to predict expression of the level of stem water soluble carbohydrates over locations, but without using explicitly measured environmental variation. The addition of remote sensing data appeared to make the greatest contribution to improving environment-specific prediction. In certain cases it has also been shown that predictions based on phenotypic data by remote sensing can perform better than marker and pedigree based predictions (Krause et al., 2019).

In the current study, we aimed to test the idea that capturing the environmental variation within and across locations with phenomic and explicit environmental data in large-scale field trials can improve yield predictions when compared with genomic data alone. Forty-four winter wheat populations comprising 2992 F2:F4 lines were created from crossing elite commercial parental lines from four breeding companies. These were tested in yield plots at two locations in two consecutive years. For each plot trait data were collected at several developmental stages, and grain yield was measured at maturity. With the collected datasets, we tested the power to predict yield using combinations of phenotypic traits (“phenomic prediction”) alone, and in conjunction with marker data as in conventional genomic prediction. In practice, commercial breeding programmes involve large-scale yield trials, which are used to train and validate genomic selection models. This study shows how phenomic selection, based largely on hyperspectral remote sensing, can be combined with genomic selection at such a scale, applied to large number of elite crosses, and which models show the best prediction accuracy.

## Materials and methods

### Germplasm

Thirty-nine bi-parental and five three-parental cross populations were used to develop 2992 F_2:4_ lines (68 per cross). The majority of this population has been described previously by Edwards et al. (2019), although an extra 11 crosses were added at a later date to our study after they became available for use. The parents of these populations were elite breeder’s germplasm consisting of both hard and soft winter wheat cultivars adapted to the United Kingdom. A total of 27 parents were used, of which eight parents were used in five or more crosses, six parents were used in three or four crosses, and the remaining 13 parents were only used in a single cross. Previous modelling studies showed that this number of crosses provided the best balance between optimising the accuracy of prediction models, costs of genotyping and phenotyping and managing the logistics of conducting large field trials (Gaynor et al., 2017).

### Genomic data

The F_2:4_ lines were genotyped using the Wheat Breeders’ 35K Axiom array (Allen et al., 2017). The DNA for genotyping was obtained by bulking leaves from approximately six F_4_ plants per F_2:4_ line. Genotype calling was performed using the Axiom Analysis Suite 2.0 with a modified version of the “best practices” workflow. QC threshold was reduced to 95 (97 normally), plate pass percent was changed to 90 (95 normally), and average call rate was changed to 97 (98.5 normally). Thresholds were reduced to accommodate the bulk sampling and early generation nature of the material used. After quality control, a total of 35,143 markers were brought forward with 13,791 markers for which codominant scores could be assigned to pools of offspring lines. Sporadic missing data were imputed using Beagle 3.3.2 (Browning and Browning, 2007). Marker sets were filtered for PIC values >0.1 (9,743 markers). Non-redundant marker datasets were created by removing markers with a correlation of >0.9 with other markers (4,404 markers).

### Trials setup

The F_2:4_ lines and agronomic checks were evaluated in 1.7 × 4 m plots at two locations (Cambridge, UK and Duxford, UK) in the 2015–16 growing season, and two locations (Duxford, UK, and Hinxton, UK) in the 2016–17 growing season (Table 1). Trials were sown between 23rd October to 4th November 2015 and 12th and 26th October 2016, and harvested between 31st August to 9th September 2016 and 15th to 29th August 2017. The preceding crop for all four trials was winter beans (*Vicia faba* L.). The soil at the Duxford (Dux) sites in 2016 and 2017 (on neighbouring fields) was a sandy clay loam in the Soham series with a clay loam subsoil overlaying chalk rubble. The Cambridge (Cam) site was clay loam (St Lawrence series) overlaying clay, and Hinxton (Hinx) was freely draining lime-rich loamy soil overlaying chalk rubble. All locations were managed for optimal yield by supplying fertilizer and applying pesticides to control disease. All 44 F_2:4_ populations were evaluated in four plots across 2 years. However, eleven of the populations were only planted in the 2016–17 growing season due to lack of availability of seed. To accommodate the missing populations, the allocation of F_2:4_ lines was highly unbalanced across years and locations as described below.

**TABLE 1:** Trial design summary showing number of replicated plots per tested line per location.

	2015/2016		2016/2017	
No. Lines	Cambridge	Duxford	Duxford	Hinxton
367	2	1	1	0
381	2	1	0	1
381	1	2	1	0
367	1	2	0	1
748	1	1	1	1
748	0	0	2	2
Total plots	2,992	2,992	2,992	2,992

The experimental design for both locations within a year was a modified α-lattice design (Patterson and Williams, 1976). The modified design consisted of a traditional, replicated *α*-lattice design with un-replicated entries added to the sub-blocks. The replicated portion of the *α*-lattice design was composed of the agronomic check varieties and half of the entries (34 of 68 lines) from 22 of the F_2:4_ populations. These entries were planted in two blocks split into 151 sub-blocks each containing five entries. The remaining F_2:4_ lines were randomly allocated to sub-blocks, bring the total number of entries per sub-block to either nine or ten. The half of the F_2:4_ entries used for the replicated portion of the design differed between locations. Thus, entries from 22 of the F_2:4_ populations were evaluated in three plots split across both locations, and the entries from the remaining populations were evaluated in two plots split across locations within a year (Table 1). Yield and grain moisture data were automatically collected by the harvester combine. Raw yield data were adjusted to 85% dry matter using measured grain moisture contents.

### Weather data

Weather data were collected on the NIAB meteorological station, Cambridge UK. For comparison of weather variables across years, daily average air temperature (°C) and rainfall (mm) were used.

### Soil measurements

To describe spatial variations in soil physical characteristics, measurements of apparent soil conductivity were made at two depths, 0–50 cm (‘Shallow”) an 50–150 cm (“Deep”). Measurements were carried out using an electromagnetic induction meter (Dualem-1S, Dualem Inc., Milton, ON, Canada) by experienced field personnel (SOYL Precision Crop Production, Newbury, UK). The vehicle pulling the sensor travelled at a speed of 6-7 kph on wheelings at the ends of yield plots, at a read rate of 1 Hz, equivalent to taking recordings every 2 m. The entire trial area was surveyed in sweeps along each row of plots. Raw data points were used to make an interpolated map of the trial site which was segmented into 0.5 m^2^ grids. Grid values falling within the boundaries of the plots, as defined by a shape file, were averaged to assign a value to each plot. In 2016, measurements were made at each trial location in late April after soil profile recharge with winter rainfall, and prior to stem extension of the crop plants. In 2017, measurements were made in the beginning of May, when some net removal of soil moisture by the crop had already occurred.

### Collection of multispectral and hyperspectral data

Hyperspectral reflectance data of the trials fields were collected via piloted aircraft at low altitude (2Excel Aviation Ltd., Northampton, UK) on a single flight per season post-flowering (27 June 2016 and 7 July 2017). Hyperspectral reflectance data (400–2,500 nm) in 3.26 nm (up to 1,000 nm) to 10.90 nm (1000–2500 nm) bandwidths were collected using Hyspex VNIR-1800 and SWIR384e cameras (Neo, Norway) mounted on a Navajo aircraft. To produce plot-level georeferenced vegetation indices (Supplementary Table S2), raw hyperspectral data were radiometrically calibrated and then atmospherically corrected to reflectance using in-scene targets placed on the ground at trial sites prior to flyover, or, when these were unable to be deployed, using a standard QUAC algorithm. Water absorption bands (>1,000 nm), which were not used for indices, were removed to reduce file sizes.

High resolution RGB images were used to create digital surface and terrain models, from which estimates of crop height were derived. Multispectral data were collected by sequential unmanned aerial vehicle (UAV) flights at 3–4 week intervals between April-September 2016 (PrecisionHawk Inc., Raleigh, NC, United States), and April-July 2017 (Environment Systems, Ltd., Aberystwyth, UK). The raw reflectance data were collected, pre-processed and computed as various VI by experienced personnel employed by the suppliers. A list of VI collected from both UAV and plane flights are in Supplementary Table S1. Redundant VIs that produced the same information were omitted. Approximately 50 unique wavebands were used, combined in different ways to compute the VIs. Best linear unbiased estimates (BLUEs) for each line and location were calculated using mixed linear models, taking into account block and subblock as random effects using R package lme4 (Bates et al., 2015). With one exception in June 2017, UAV measurements were made at both trial locations on the same day.

### General crop assessment

During the growing season simple checks were performed on the all plots to identify any agronomic issues, to ascertain when hyperspectral and multispectral data collection should begin and to provide a ground based reading to compare against remote sensing data. Emergence and early season crop cover were scored visually for the 2016/17 season to identify any drilling issues. When disease was present in the trial a general disease score (0 = no disease, 9 = >90% of plants with symptoms) was assigned to each plot. All plots were visually scored for growth stages (Zadoks et al., 1974) at three different time points to decide when best to schedule UAV flights to coincide with the start of flowering (GS 61) in the majority of lines. The EARLY time point coincided with the majority of lines being at GS 39 (flag leaf emergence) whilst MID and LATE time points coincided with GS 55 (half of the ear emerged) and GS69 (end of flowering), respectively (Supplementary Table S1). During grain filling, the level of senescence (0 = no senescence, 9 = fully senesced), presence/absence of awns, waxiness (0 = no glaucousness, 9 = densely glaucous) were scored visually. Plant height was measured as average height of the canopy from three different points per plot.

### Yield predictions

Yield predictions were performed using as covariate predictors either phenomics trait data, marker data or both, using elastic net regression (R package elasticnet; Zou and Hastie, 2005). Lasso and ridge regression (both R package glmnet) methods were also compared. The models were trained on randomly selected subsets of either raw plot values or line BLUEs on each location and tested on the remaining plot values or BLUEs. Fraction ratios of training vs. test sets were experimentally varied. Resampling strategies were repeated 100 times. The effect of the quadratic penalty parameter (lambda) setting was manually evaluated and set to 0 for lasso regression, 0.1 for elastic net regression and 1 for ridge regression. The lambda value of 0.1 was chosen after it was established in preliminary analyses that in a range of 0.1–0.9 in steps of 0.1, this setting resulted in the highest prediction accuracy after cross-validation. Each variable combination in which resampling was involved was repeated 100 times. Multivariate genomic prediction was done by multivariate GBLUP implemented in R package AlphaMME (https://bitbucket.org/hickeyjohnteam/alphamme/) using as covariates either the first three principal components (R package adegenet; Jombart, 2008) over all non-yield trait data, or the three or four largest PLS regression coefficients (R package pls; Liland et al., 2016) with yield as the response variable. Predictions were based on marker and/or trait data only; relationship information for the lines was not used, although full pedigree data were obtained (Fradgley et al., 2019). Prediction accuracies were calculated as the squared correlation between predicted and observed values.

## Results

### Data set characteristics

**FIGURE 1:**
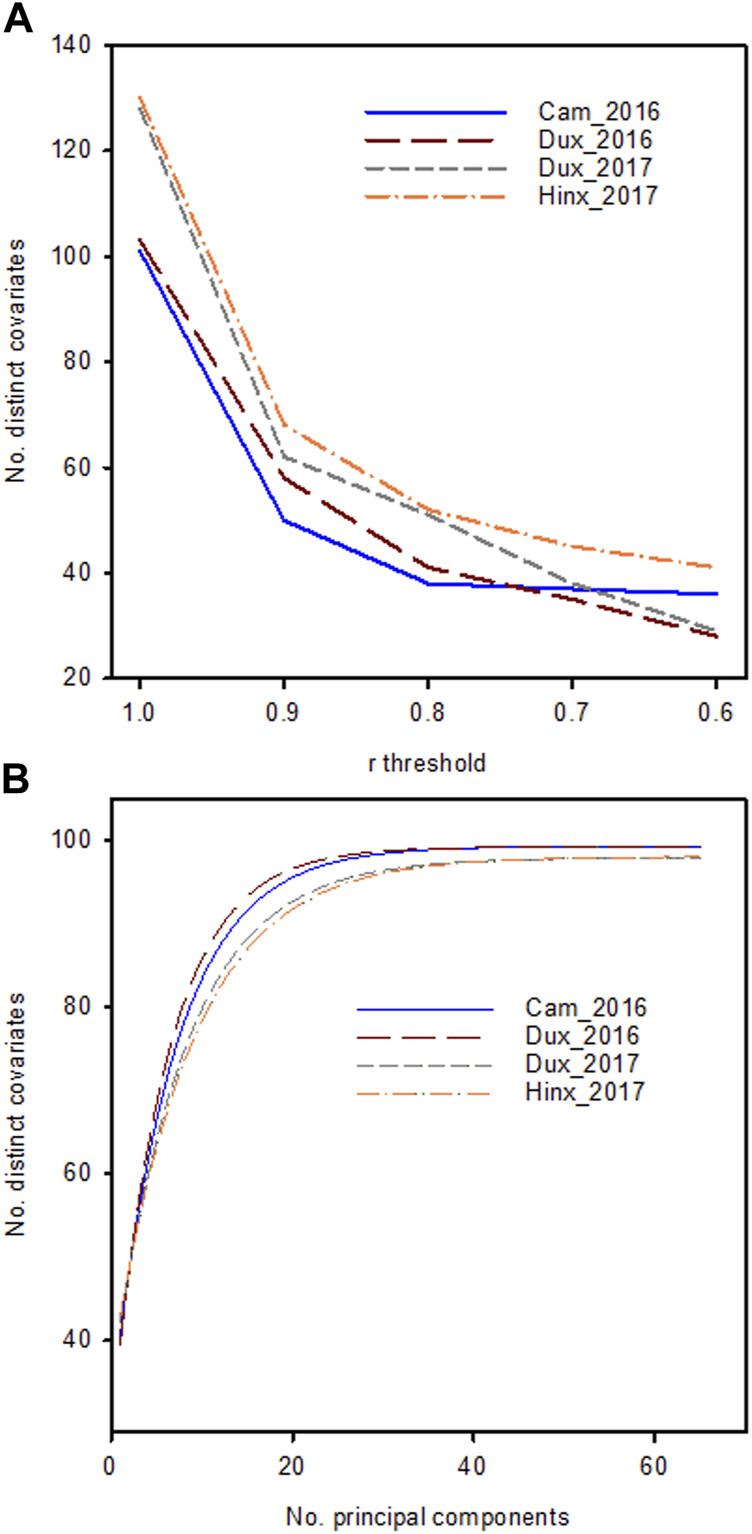
**(A)**: Number of unique traits in the phenomic data set in the four location/year combinations with mutual correlations lower than the threshold on the *x*-axis **(B)**: Cumulative explained variance as a function of the number of principal components included. Lines are fitted to data using a spline curve fitting procedure.

The current study was conducted on data collected on a large winter wheat panel that was created specifically for this research. For this program, 44 wheat populations were created from a total of 27 parents (Edwards et al., 2019), from which 2,992 lines were tested in yield trials at four locations in the years 2015/2016 and 2016/2017 (Table 1). In addition to grain yield, phenomic data comprised: ground-based visual scores; measurements of soil physical properties; vegetation indices VI) calculated from multispectral reflectance data collected with unmanned aerial vehicles (UAVs); and from hyperspectral reflectance data collected using piloted aircraft. In total 104 trait variables (including yield) were collected in the 2015/2016 trials and 130 traits in the 2016/2017 trials (Supplementary Table S1). VI produced by multispectral and hyperspectral data in general were highly inter-correlated because of the similar wavebands used for calculations or because different wavebands of the spectral reflectance were related to similar physico-chemical aspects of the crop. After eliminating redundant VI that produced the same information, the number of non-redundant, distinct traits was approximately 40 on both years (Figure 1). Even though individual reflectance wavebands from the hyperspectral measurements were not used here, the high dimensionality of the data captured by principal component analysis (Figure 1A) and the small decay in correlation threshold as redundant trait covariates were eliminated (Figure 1B) indicated that approximately 40 distinct traits effectively captured the variation without overfitting. Similarly, Zhu et al. (2021) found in soybean that reducing the number of wavebands did not weaken predictive ability. A reduction in the number of variables that needs to be considered in a prediction model is akin to the selection of tagSNPs through elimination of correlated alleles from neighbouring SNPs (Pettersson et al., 2009).

**FIGURE 2:**
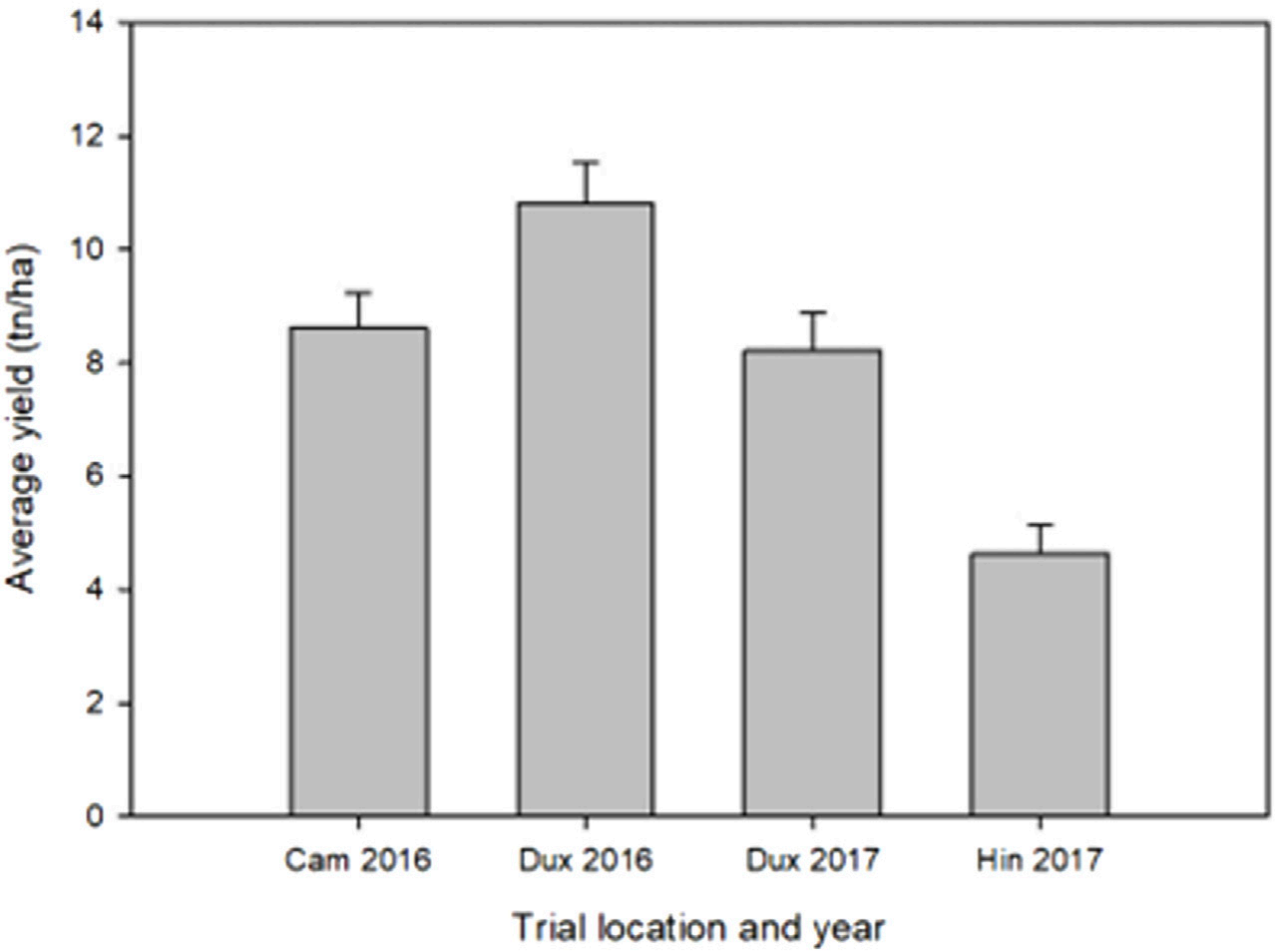
Average grain yield for each environment. Error bars indicate standard deviation.

The yield data showed large variance among the four trials due to environmental differences between sites and years. The greatest average yield was observed at Duxford in 2016 and the smallest at Hinxton in 2017 (Figure 2). Cumulative precipitation was greater during the growing season 2015/2016 (620 mm) than in 2016/2017 (583 mm). Yields at the Hinxton site in 2017 were affected by lighter soils (sandy loam) and dry conditions during a period without rainfall from 17 April to 2 June 2017, with only 42 mm recorded between 4 March and 15 May 2017, compared with the long-term average (1986–2016) of 59 mm and 108 mm during the same period in 2016. During that period, the only day with significant (>5 mm) rainfall occurred on 22 March 2017 (7.5 mm). The accumulated soil moisture deficit (ETo minus rainfall) of 152 mm, which reached a maximum on 26 June 2017, was accompanied by delayed N uptake, as fertiliser remained on the soil surface until it was solubilised by rainfall that re-commenced 16–18 May 2017 (48 mm). The water holding capacity (to an effective rooting depth of 1.2 m) at the Duxford sites was 187 mm, 199 mm at Cambridge, and 198 mm at Hinxton. These conditions are not unusual for wheat production in the region. Low rank correlations for yield for lines shared across sites were observed among the four locations, indicating strong GxE effects between trials (Table 2). In 2017, air temperatures increased a week earlier in spring compared with 2016; however at harvest time, all trial fields had been exposed to the approximately same growing degree days (range 2,527–2,610 °Cd).

**TABLE 2:** Spearman’s rank correlations for yield among shared lines between locations.

	Cam 2016	Dux 2016	Dux 2017
**Dux 2016**	0.389	1	
**Dux 2017**	0.233	0.275	1
**Hinx 2017**	0.150	0.192	0.352

### Phenomic yield prediction

**FIGURE 3:**
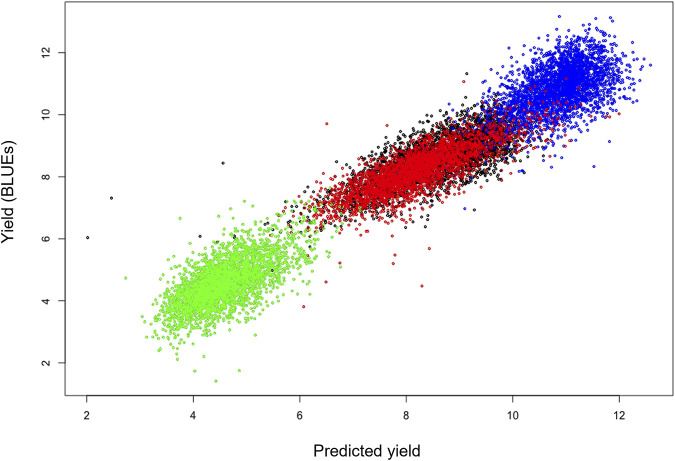
Lasso regression fits for yield using the phenomics data as covariates for trials within a year. Scatter plots show predicted yields (horizontal axis) for each site using a model trained on phenomics data across both locations, vs. observed yield data (vertical) for individual plot yields. R^2^ values indicate the goodness of fit. Colour coding: Blue: Cam 2016 (R^2^ = 0.62); Red: Dux 2016 (R^2^ = 0.37); Green: Dux 2017 (R^2^ = 0.79); Black: Hinx 2017 (R^2^ = 0.76).

As a first characterization of the suitability of the phenomics traits as predictive covariates for yield, lasso models were created with data from both locations within a year and model fits were compared to the original data (after rescaling to the original scales per location; Figure 3). The models showed a high fitting accuracy with no bias over both locations within a year, except for Duxford 2016 for which the overall fit was lower than that of the other three locations. The variation in the phenomics data explained most of the variation in yield, suggesting that all or part of the phenomics data would be good proxies for yield. Inspection of the fitted lasso coefficients showed that the highest contribution were from the multi- and hyperspectral data, while the lowest coefficients were observed for the soil measurements and the ground-based visual scores.

To test the power of the phenomics data to predict the validation data, various fractions of the total set of plots (1, 0.75, 0.5, 0.25, 0.15, 0.10, 0.05, 0) in both locations of both years were kept for the training phase using lasso, elastic net or ridge regression models, and the rest were used as a validation set (Table 3, Supplementary Table S2). Again, we observed high prediction accuracies. In general, the accuracy for a location only dropped when less than 10% of its entries were included in the model. Elastic nets was clearly less sensitive to this effect than lasso regression. The former method shows slightly lower overall prediction accuracy because it balances the predictive power between selected covariates with a detectable individual effect and spreading the variation evenly over all covariates. All predictions were more accurate for the 2017 trials than for the 2016 trials. Repeating all analyses using line corrected averages (BLUEs) yielded similar trends as the analysis using uncorrected averages (on a per plot basis), except that all accuracies were on average 0.046 lower for the 2016 data and 0.097 lower for the 2017 data. Trends from the ridge regression analysis were similar to those from elastic net, except that all accuracies of ridge regression were on average 0.02–0.03 smaller (not shown).

**TABLE 3:** Selection of phenomic yield prediction accuracy for random masked data for two locations within a year. Prediction results are from raw plot yield data and for BLUEs using the Lasso and Elastic net regression methods. For full summary of phenomic yield prediction accuracy see Supplementary Table S2.

		2016				2017			
Training fractions		LASSO	Elastic Net	LASSO	Elastic Net	LASSO	Elastic Net	LASSO	Elastic Net
Location 1	Location 2	RAW	RAW	BLUE	BLUEs	RAW	RAW	BLUE	BLUEs
1	1	0.53	0.46	0.49	0.45	0.78	0.74	0.68	0.65
1	0.5	0.38	0.31	0.36	0.33	0.72	0.70	0.66	0.64
1	0.1	0.32	0.29	0.33	0.31	0.67	0.65	0.60	0.60
1	0	0.22	0.27	0.22	0.30	0.50	0.59	0.47	0.57
0.5	1	0.62	0.58	0.56	0.54	0.77	0.75	0.62	0.61
0.5	0.5	0.52	0.45	0.47	0.44	0.76	0.73	0.66	0.64
0.5	0.1	0.45	0.40	0.42	0.40	0.73	0.70	0.63	0.62
0.5	0	0.30	0.38	0.28	0.36	0.16	0.65	0.50	0.59
0.1	1	0.54	0.54	0.51	0.51	0.73	0.71	0.57	0.57
0.1	0.5	0.51	0.47	0.47	0.45	0.75	0.72	0.62	0.61
0.1	0.1	0.48	0.44	0.42	0.40	0.74	0.72	0.61	0.62
0.1	0	0.18	0.41	0.11	0.24	0.02	0.58	0.23	0.51
0	1	0.23	0.52	0.30	0.49	0.05	0.68	0.49	0.54
0	0.5	0.25	0.45	0.29	0.43	0.03	0.69	0.48	0.58
0	0.1	0.15	0.40	0.14	0.34	0.02	0.66	0.18	0.54

Table 3 (and Supplementary Table S2) shows that using the elastic net method a reasonably accurate yield prediction over locations is possible based only on phenomic measurements, provided that the same phenomics traits have been collected on a reference site for which yield data are known. For instance, if a prediction model for 2016 was trained on 50% of the yield data from Duxford and only 10% of the yield data from Cambridge, prediction accuracy for yield across sites was 0.5 (Table 3, Supplementary Table S2). Table 3 (and Supplementary Table S2) values are based on the average accuracy over both locations in each year; however, there are appreciable differences in goodness-of-fit among the locations, as illustrated in the scatterplots (Figure 3). There were also differences among average prediction accuracies per location over all resampling combinations. For instance, average accuracies (expressed as R^2^ values for each site) of the ridge regression predictions, which show the lowest dependency on training set size, were: 0.55 (Cam 2016); 0.30 (Dux 2016); 0.72 (Dux 2017); 0.65 (Hinx 2017). Lasso prediction accuracies for each site (Figure 3) were: Cam 2016 (R^2^ = 0.62; blue); Dux 2016 (R^2^ = 0.37; red); Dux 2017 (R^2^ = 0.79; green); Hinx 2017 (R^2^ = 0.76; black).

**FIGURE 4:**
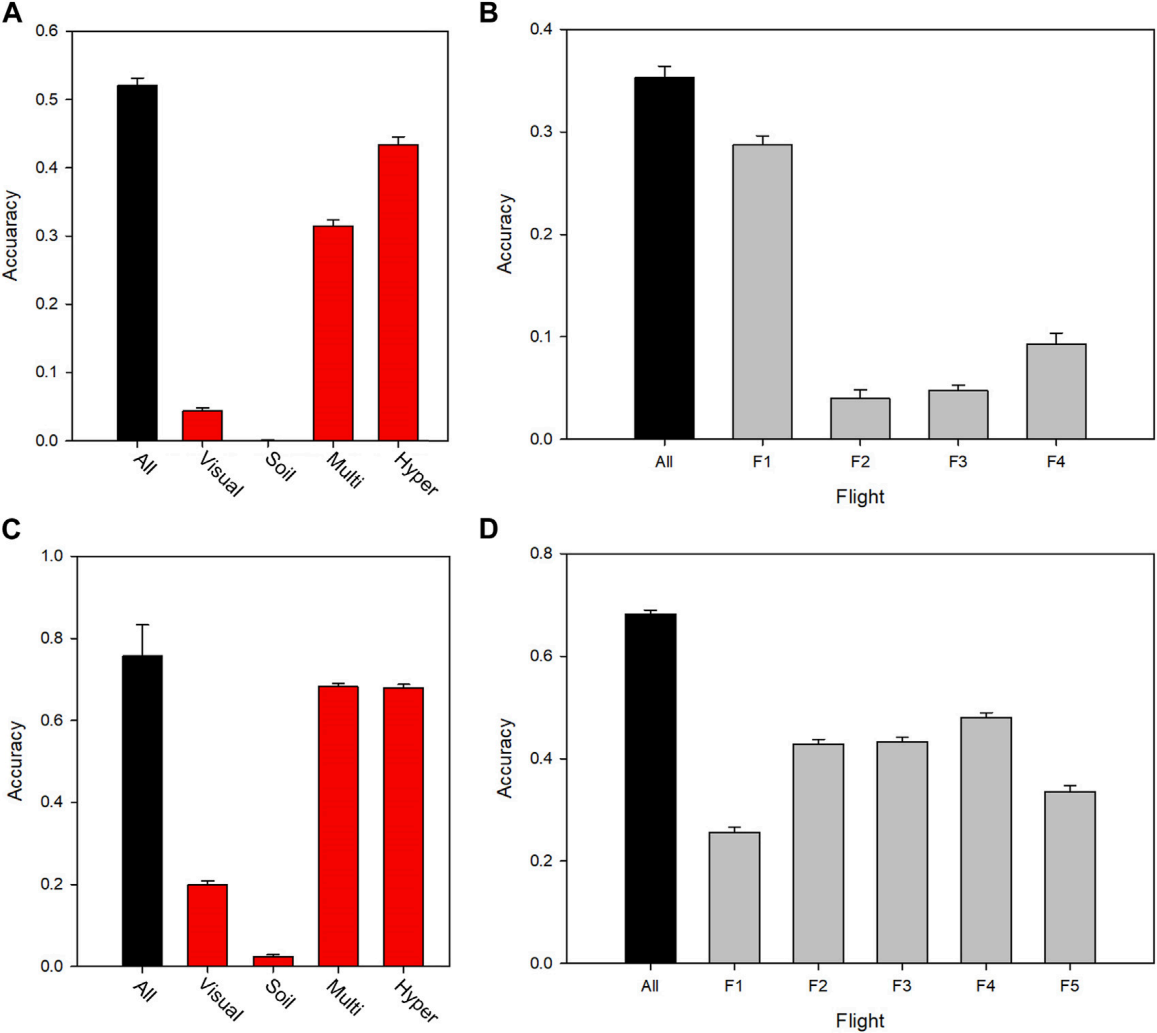
Yield prediction accuracy for different sources, types and times of measurement for 2016 (top panels) and 2017 (bottom panels), leaving out a random 50% of plots from both locations for validation of the model training set. **(A)** (2016) and **(C)** (2017) show prediction accuracy for data subsets in each year, in which traits were classified according to data type. **(B)** (2016) and **(D)** (2017) show the predictive power of the multispectral trait datasets in each year, separated by time of measurement, F1-F5 (spanning the period mid-spring until the end of summer). Supplementary Table S1 shows the dates of each flight **(F)**. Error bars indicate standard deviation over the data re-samplings.

In order to obtain a better understanding of the nature of the predictive power of a heterogeneous phenomics dataset, traits were subdivided in categories. These included the type of trait (or environmental covariate in the case of soil data) and time point of measurement (Supplementary Table S1). Figures 4A,C clearly show that the spectral data sets, which also contain most of the observations, account for the vast majority of the prediction power. For 2016, the multispectral data were less predictive than the hyperspectral data, whereas in 2017 they were similar. It is interesting to note that the traits described by the multispectral data were essentially plant height and green canopy cover (estimated in different ways with different spectral indices), whereas the hyperspectral data reflected a larger range of plant traits. This indicates two things: that a relatively small number of phenomic traits can have good predictive power, and that light interception dominates the relationship to yield. The contribution of manually assessed visual scores towards yield prediction was small, including assessments of general phenological development. The prediction accuracy of the multispectral data increased to a maximum corresponding to GS 55, and then decreased as the crop senesced (Figures 4B,D).

**FIGURE 5:**
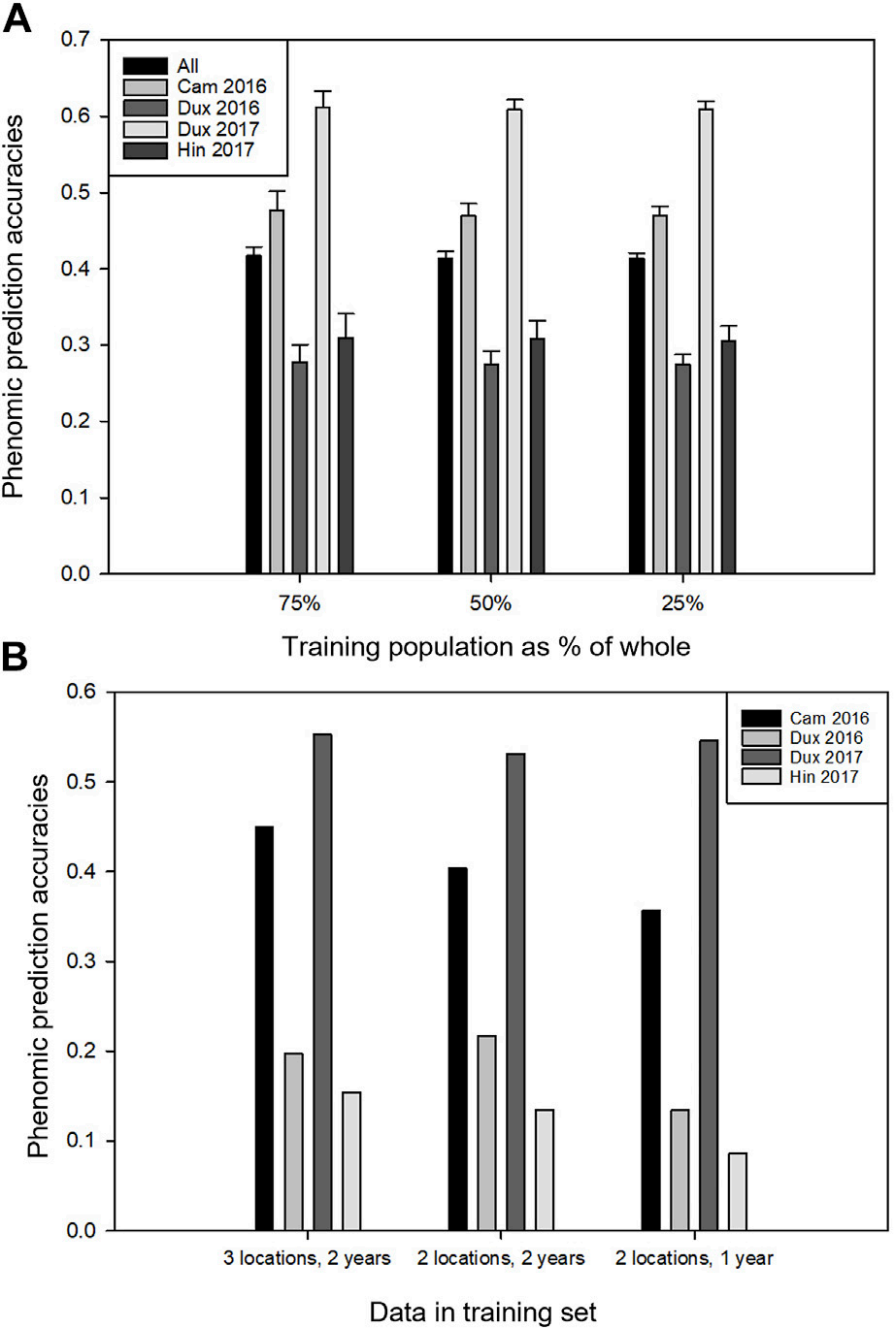
Phenomic prediction accuracies of yield using the elastic nets method, predicting over trial years and locations. **(A)** The effect on prediction accuracy at each location when different fractions of the dataset were masked (equal for each location) and the remaining plots were used for model training. Error bars indicate standard deviation over the data resamplings. **(B)** Prediction of full locations from the other locations. First series: three locations were used for training and prediction was done on the fourth location; second series: two locations in two different years were used for training and prediction was done on a third location; third series: two locations in a single year used for training, prediction of a third year.

The overall dataset was disjointed, as a set of lines part was tested only in 2017 (due to a delay in the availability of seed; Table 1), and some of the traits were not measured in both the years (Supplementary Table S1). The prediction accuracies were highly variable depending on location. As atmospheric and radiometric corrections were applied to the remote sensing data, site effects are most likely due to the biological status of the crop. Predictions for Duxford 2017 were always greater than those of other locations (Figure 5). Prediction of whole locations (e.g., from which no data have been used in the training phase) always had a lower accuracy than training sets composed of data from all locations (using test conditions with comparable size sets) (compare Figures 5A,B). Training on three full locations gave better results than training on two. Training on two full locations from two different years were better than two of the same year, indicating that best results are obtained when year-by-year variation is represented in the training set (Figure 5B). The effect of all these tendencies was dwarfed by the variation in accuracy according to location. For instance, the best phenomic prediction accuracies for whole-location prediction were consistently found for the trials in Cambridge 2016 and Duxford 2017. The results illustrate the importance of the environments used for the training sets, which must be representative of the target environments to obtain the best predictions, even when using environmental covariates along with genomic and or phenomic markers (Rogers and Holland, 2022).

In general, visual trait scores showed poor correlation with yield (r < 0.1), although plant height showed greater correlations with yield in 2017 (r = 0.3–0.4). It is unlikely this was a data quality issue; for example, leaf wax related traits were consistent over two locations, and correlated (r = 0.33–0.45) with particular hyperspectral VI. Visual scores of growth stage did not correlate well with other measurements. Manual measurements of canopy height correlated well (r = 0.60–0.67) with height estimates calculated from the digital surface models derived from the UAV RGB images.

### Combining phenomic and genomic data for prediction

A major aim of the study was to capture environmental variation to make genomic prediction over locations more reliable. Differences in genotype-specific responses to environments may erode the prediction accuracy of a model trained in one environment when it is applied to materials grown in another environment (Mackay et al., 2015; Crossa et al., 2017). As the current phenomic data have been demonstrated to have predictive power towards yield, these could be used to complement marker data to predict genotype performance in different environments.

**FIGURE 6:**
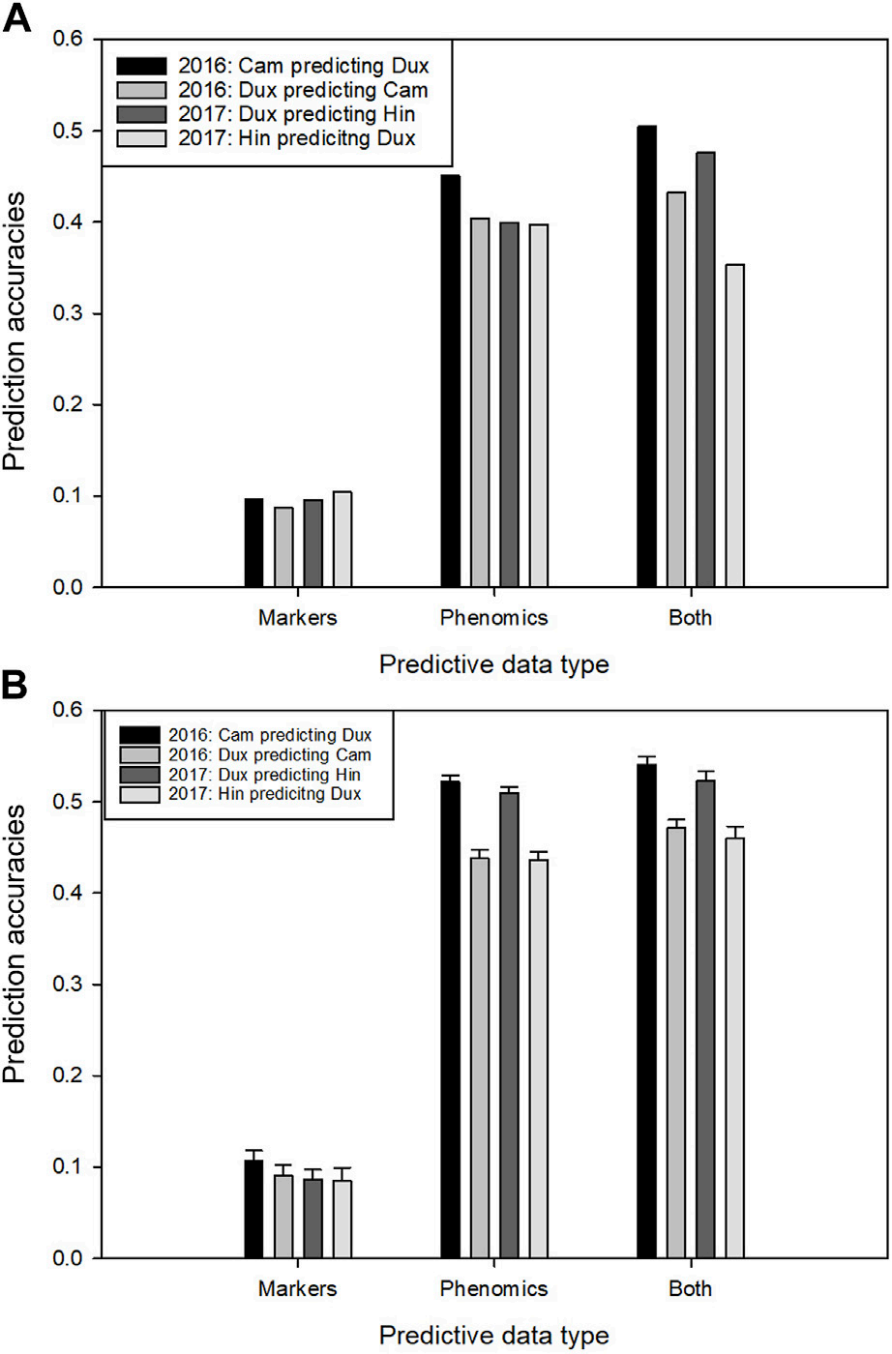
Prediction of yield for full trial locations within each trial year using two types of predictive data: markers, phenomic trait data and these two combined. **(A)** Four within-year combinations of a prediction model trained on yield line averages of one location, applied to predict yield on the other location, using marker data, phenomic data and both. **(B)** as in A, however here the training sets have been supplemented with 10% random fractions of the same data types from the test location. Error bars indicate standard deviation over the data re-samplings.

Using only the genetic marker set, the predictive power over locations (without inclusion of data of the test site in the model) was low (Figure 6A). This was expected, given the observed occurrence of strong GxE effects for yield among the four trial locations. Prediction using the phenomics data for the same combination had dramatically greater accuracy. The combined prediction power of both data types together was slightly greater than that of only the phenomics data, except for Hinxton 2017. The average improvement of the combined data models over the best single data type model (the phenomics model) was 6.7% (or 12.6% improvement when not taking in account the data for Hinxton 2017). When a small fraction (10%) of data from the test location was added to the training set, the accuracy when using the phenomics data increased, but the complementary effect increase of both data types was lower (Figure 6B). When larger fractions of data from the test location were added to the training set, the accuracies of phenomics and combined models eventually converged to the same level (not shown).

**FIGURE 7:**
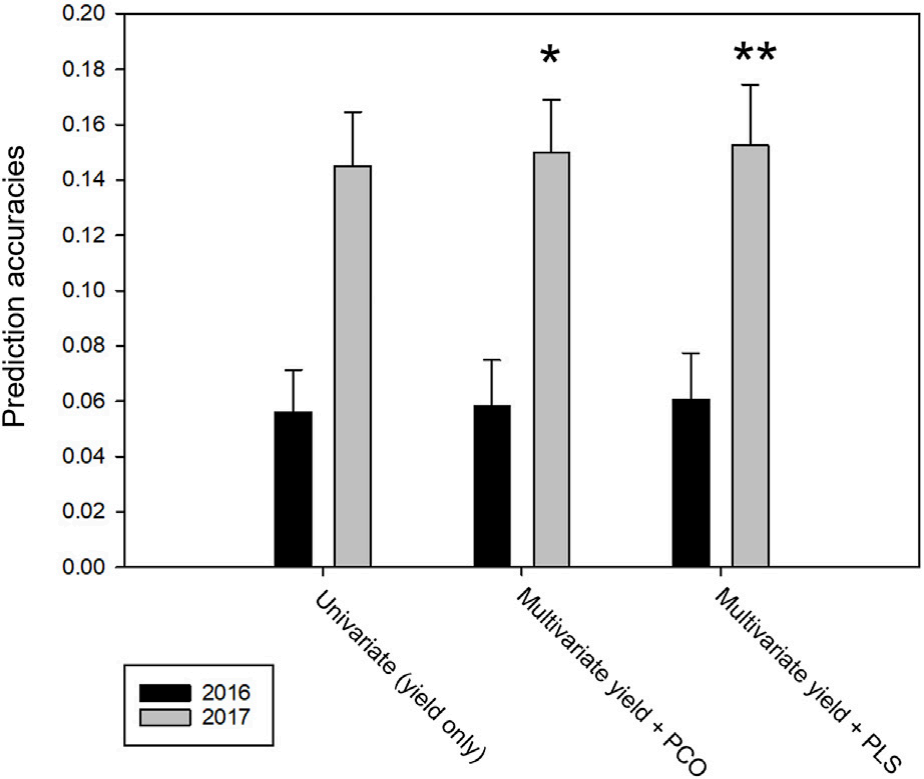
Genomic prediction accuracies using yield and phenomics data as co-response variables and markers as covariates on data of both locations within each trial year; 80% line averages randomly sampled for training, 20% for validation. Series show prediction accuracies for univariate (yield only), multivariate yield plus first three principal components of phenomics data and multivariate yield plus PLS components with highest loadings, respectively. Error bars indicate standard deviations over the data resamplings. Asterisks indicate the one-tailed *t*-test significance between univariate and multivariate accuracies.

As the dimensionality of the current phenomics datasets is too large to be included in full in a multivariate genomic prediction model and there was large cross trait correlation, we reduced phenomic trait data to either the first three principal components (PCO) or to the major components in a partial least square (PLS) analysis with yield as response variable. With PCO, taking in consideration the loadings in the various sources of the phenomics traits, we selected the four components with the highest loadings for the 2016 trial data and the three components with highest loadings for the 2017 trial data (Table 4). In this setup, multivariate predictions were done using data from both locations within each trial year, with randomly masked line average yields used as training and the remainder for validation. The addition of the PCO components as response variables had no effect on prediction accuracy in the 2016 data, while there was a modest improvement in prediction accuracy in the 2017 data. In the latter case, the inclusion of the yield related PLS components had a larger effect than the addition of the neutrally selected PCO components (Figure 7). When the model was trained on single locations (e.g., predicting yield performance on all lines at the second location), no change in prediction accuracy was found for multivariate over univariate analysis in any combination of locations and components.

**TABLE 4:** Selected components analysis to reduce the dimensionality of the phenomics data. For each trial year (combining both sites), the first three principal components (PCO) are shown as well as the components with the highest loadings in a Partial least squares regression (PLS analysis) with yield as the response variable. For the PLS components, the trait categories with the highest loadings are indicated X). See text for dates and approximate growth stages when measurements were taken.

Trial year	Component	Correlation to Yield	Main composition (PLS loadings)			
			Early multispectral	Late multispectral	Hyperspectral	Field scores
**2016**	PCO 1	0.16				
	PCO 2	0.14				
	PCO 3	−0.04				
	PLS 1	0.47	X		X	
	PLS 2	0.35	X	X	X	
	PLS 3	0.21		X	X	
	PLS 4	0.17	X		X	X
**2017**	PCO 1	0.17				
	PCO 2	0.11				
	PCO 3	0.040				
	PLS 1	0.63		X	X	
	PLS 2	0.35	X	X	X	
	PLS 3	0.25			X	X

## Discussion

In this study we tested the idea that phenomics data collected on wheat yield trials could capture the component of yield variance due to environment variation, predict yield across location and years, and potentially complement genomic prediction. For this purpose we collected more than 100 different traits using different remote sensing methods and traditional ground-based visual assessments. As ‘pure’ environmental covariates, physical soil attributes were collected at the plot level as well. A similar approach has been reported by Rutkoski et al. (2016) with more trial locations but a limited amount of phenomics traits. To our best knowledge, the combination of the wide range of phenomic data collected on >12,000 plots across four locations over 2 years, marker data on >3,000 elite crosses from within commercial breeding programmes, makes the current study one of the largest public wheat study of its kind so far.

Hyperspectral imaging has been used extensively for plant phenotyping, as the signatures of reflected light across a broad spectrum from plant surfaces can be related to a wide range of physiological features, and has been used to predict grain yield in some studies (Sarić et al., 2022). A large amount of data can be collected using hyperspectral analysis when considering the spatial distribution of the waveband stacks for each image pixel, but our correlation analysis showed that large proportions of variation were redundant over the traits. In the total dataset across all data sources, the effective number of non-redundant traits was consistently found to be around 40. From the total variation present in all traits, 50%–60% could be explained by the first three principal components. In contrast, a study by Aguate et al. (2017) found that using individual hyperspectral wavebands in the phenomic prediction model of maize yield performed better than vegetation indices.

Spectral data collected during mid-grain fill (approximately 15 days after flowering) had strong predictive power for yield in the current datasets, both within location and over locations. Similar results have been reported in other studies (Das et al., 1993; Yue et al., 2021). Detailed analysis of the multispectral data collected by UAV at various time points during the season suggests that the predictive power of these data types initially increased over time to flowering/grain fill stage but then decreased at the ripening stage. This is unsurprising as the majority of VIs focus on chlorophyll-related traits (e.g., canopy cover, photosynthetic activity), whilst loss of chlorophyll in a senesced crop will have diminished the discriminatory utility of the VIs. In future work, the time-course data can be analysed further to derive longitudinal variables that describe dynamic changes in the crop, such as rates of canopy expansion and senescence, which are affected by genotype and environmental conditions. Quantification and modelling of these dynamic changes have recently been shown to be good predictors of wheat yield (Fu et al., 2020) (Sun et al., 2022). Similar time-course data have been incorporated into gene models (Bustos-Korts et al., 2019). As the costs of a single flight via piloted aircraft for hyperspectral imaging is comparable to multispectral (UAV) flights on multiple dates, both approaches in our study offer similar prediction power for the money spent. However, more recently modelling dynamic changes in crop development over numerous UAV-multispectral flights offer increasingly better predictions of yield (Fu et al., 2020), then the UAV-multispectral option would prove most appealing in breeding settings. A number of morphological features, such as height, lodging susceptibility, ear emergence, etc., can be derived using a relatively low cost RGB camera, precluding the need for a more expensive multispectral sensor (Sun et al., 2022). From the current observations, it is difficult to draw conclusions about the biological basis of the various components of spectral reflectance that contributed to the prediction of yield, but this lack of fundamental understanding does not preclude use of the phenomic data as predictors. Other studies have shown that spectral-based phenomic selection can be as good as or better than genomic selection for yield prediction, including near-infrared spectroscopy of grain or flour samples as predictors (Robert et al., 2022).

Ground-based, visual crop assessment scores made little contribution to the accuracy of the yield predictions, including assessments of genotypic differences phenological development. These findings imply that remote sensing techniques cannot, at present, simply be used to directly replace traditional visual scoring methods used currently in wheat breeding and research programs. A more reasonable assumption would be to use remote sensing data to complement current techniques by collecting data on valuable traits that are difficult to score visually, such as nitrogen or water content (recently reviewed in Blatchford et al., 2019; Fu et al., 2021).

Environmental covariates (either weather-derived or generated using crop models) have been incorporated into G x E prediction models to improve the accuracy of genomic selection across environments (Jarquin et al., 2013; Heslot et al., 2014; Saint Pierre et al., 2016), but not in all cases (Widener et al., 2021). With only four environments in our study, such an approach would not have been effective. Instead, we attempted to capture within trial environmental variation by measurements of apparent soil conductivity (measured at two depths) at meter spatial resolution so that covariates could be assigned to each plot. Measurements of soil nutrients were also interpolated to the individual plot level. The soil conductivity measurements were expected to be a proxy for local variation in water holding capacity, which could have affected yield in a dry season. It was anticipated that these covariates would account for a portion of the environmentally-induced variation in observed yields, as found by Vieira et al. (2022). However, despite variation in soil conductivity across the fields in each trial, no detectable relationship to the yield variation was found. It could be that the levels of spatial differences measured with this technique were not sufficiently large enough to have a detectable effect on yield. Recently, a study that combined explicit, known environmental variables with latent variables achieved improvements in predictive power of a multi-location genomic selection model (Tolhurst et al., 2022). Although in our study the four environments were too few to adopt this strategy, this provides encouragement that collection of explicit environmental data at test locations can be beneficial.

While the predictions based on the phenomics data were in general accurate and robust, this was not the case for predictions based on the marker data. We noted that all marker-based prediction accuracies are lower than previously observed in wheat (e.g., Heslot et al., 2014; Lopez-Cruz et al., 2015; Battenfield et al., 2016; He et al., 2016; Rutkoski et al., 2016; Norman et al., 2018). A possible explanation is that the markers for this study have been assayed on pools of F2:4 offspring, which might induce an extra level of uncertainty on the genotype scores.

The complementary predictive power of the phenomics data with the genomic data was found to be low, suggesting that the genotypic variance is almost fully covered by the phenomics traits. The level of complementary effect is in the same range found by Heslot et al. (2014) when applying explicit environment-crop interaction models, but smaller than that found by Lopez-Cruz et al. (2015) with the application of marker-by-environment interaction models (without use of explicitly measured data to represent the environmental variation). Rutkoski et al. (2016) showed that modelling three remote-sensing covariate traits increased prediction accuracy substantially; however, the predictive effect of the remote sensing data without markers was not included in this paper, so the complementarity of both data types cannot be inferred from the results. The improvements in the accuracies by inclusion of the remote sensing data reported by Rutkoski et al. (2016) were in general lower than we found with the data in this study. Crain et al. (2018) found that including two remote sensing traits with genomic predictions gave small improvements for most of the investigated trial combinations.

The use of marker data alone for genomic selection is appealing to breeders, as genotyping is relatively easy and cost-effective, and can even be conducted on seed materials without the need to grow the actual plants for which the traits are to be predicted (Lorenz et al., 2011). In our study, phenomic data were collected on large plots that were also used to measure yield. Additionally, we demonstrated that multivariate prediction of yield together with representations of the measured phenomics variation resulted in very small improvements over univariate prediction of yield, as has previously been reported by Rutkoski et al. (2016). This would be a logistically and financially demanding strategy, and as yield is already being measured as part of a yield trial there is little value in a prediction model. However, the phenomics data prediction accuracy when compared to the genomic predictions in this study show great potential in terms of prediction accuracy towards yield in winter wheat; Krause et al. (2019) showed similar findings when comparing hyperspectral based phenomics verses marker and pedigree predictions. However, in our study the predictive accuracy of the phenomic data was greater, relative to the marker data, than that shown by Krause et al. (2019). Whilst unlikely to be used on yield trials, a practical application of this approach might be to train and use the model to test yield potential of family breeding materials grown in small plots or ear rows that are too small for accurate yield measurement but can be measured for phenotypic traits using high resolution remote sensing methods. Current advancements in low-cost remote sensing technology now allow this (Sun et al., 2022). For example, our observations in the 2017 trial suggest that even predictions based on relatively cost-effective multispectral measurements made more than 4 months before the harvest date can be twice as accurate as genomic prediction.

The concept of genomic prediction is that all materials are genotyped because genomic data are relatively cheap to obtain from seed or seedlings and only a fraction of the materials are measured for yield, which is relatively expensive. Our original idea was that low-cost, highly dimensional phenomic markers could be used in the same sense as genomic markers and creating a richer dataset in combination. Surprisingly, the results show that prediction algorithms based on a relatively small number of phenomic markers alone are as good or better than a large set of genomic markers or the set of combined markers. This suggests that individual phenomic markers have greater intrinsic value than individual genomic markers although individual markers of any sort explain only a small fraction of the variation in yield. For example, a single phenomic marker that corresponds to green canopy cover is genetically determined by a large number of genes acting in concert to produce that phenotype. Alternatively, a single SNP may have a tiny effect on any observable phenotype. Furthermore, the phenomic marker, as a measure of the expression of multiple genes under certain environmental conditions, also encapsulates epigenetic effects that cannot be readily described by markers based on DNA sequence alone. Where efforts to use phenomic markers have failed in the past to gain acceptance in selection programmes might have been due to the limited number of markers that were employed, and the time and cost required to collect those phenomic data. Techniques are now available to vastly increased the number of phenomic markers and decrease acquisition costs (Sun et al., 2022). The results of this study show how such datasets could be used and their value for yield prediction across sites and years.

## Data Availability

The original contributions presented in the study are included in the article/Supplementary Material, further inquiries can be directed to the corresponding author.
