# Supplementary material for: Phenomic and genomic prediction of yield on multiple locations in winter wheat
Source: Front Genet. 2023 May 9;14:1164935. doi: 10.3389/fgene.2023.1164935 (PMC10203586; doi:10.3389/fgene.2023.1164935)
Supplement: Supplementary file 1 [file Table1.DOCX]

**Supplemental Table 2:** Phenomic yield prediction accuracy for random masked data for two locations within a year. Prediction results are from raw plot yield data and for BLUEs using the Lasso and Elastic net regression methods.

|  |  | 2016 | | | | 2017 | | | |
| --- | --- | --- | --- | --- | --- | --- | --- | --- | --- |
| Training fractions | | LASSO | Elastic Net | LASSO | Elastic Net | LASSO | Elastic Net | LASSO | Elastic Net |
| Location 1 | Location 2 | RAW | RAW | BLUE | BLUEs | RAW | RAW | BLUE | BLUEs |
| 1 | 1 | 0.53 | 0.46 | 0.49 | 0.45 | 0.78 | 0.74 | 0.68 | 0.65 |
| 1 | 0.75 | 0.39 | 0.32 | 0.36 | 0.33 | 0.75 | 0.71 | 0.68 | 0.65 |
| 1 | 0.5 | 0.38 | 0.31 | 0.36 | 0.33 | 0.72 | 0.70 | 0.66 | 0.64 |
| 1 | 0.25 | 0.36 | 0.30 | 0.35 | 0.32 | 0.70 | 0.67 | 0.64 | 0.62 |
| 1 | 0.15 | 0.34 | 0.29 | 0.34 | 0.32 | 0.69 | 0.66 | 0.62 | 0.61 |
| 1 | 0.1 | 0.32 | 0.29 | 0.33 | 0.31 | 0.67 | 0.65 | 0.60 | 0.60 |
| 1 | 0.05 | 0.30 | 0.28 | 0.32 | 0.31 | 0.65 | 0.63 | 0.57 | 0.59 |
| 1 | 0 | 0.22 | 0.27 | 0.22 | 0.30 | 0.50 | 0.59 | 0.47 | 0.57 |
| 0.75 | 1 | 0.64 | 0.58 | 0.58 | 0.54 | 0.78 | 0.76 | 0.64 | 0.62 |
| 0.75 | 0.75 | 0.53 | 0.45 | 0.47 | 0.44 | 0.76 | 0.73 | 0.66 | 0.64 |
| 0.75 | 0.5 | 0.48 | 0.40 | 0.44 | 0.40 | 0.75 | 0.72 | 0.67 | 0.64 |
| 0.75 | 0.25 | 0.44 | 0.38 | 0.41 | 0.38 | 0.74 | 0.70 | 0.65 | 0.63 |
| 0.75 | 0.15 | 0.42 | 0.36 | 0.39 | 0.37 | 0.72 | 0.69 | 0.63 | 0.62 |
| 0.75 | 0.1 | 0.40 | 0.35 | 0.39 | 0.36 | 0.71 | 0.68 | 0.62 | 0.61 |
| 0.75 | 0.05 | 0.38 | 0.35 | 0.37 | 0.36 | 0.68 | 0.66 | 0.59 | 0.60 |
| 0.75 | 0 | 0.28 | 0.34 | 0.28 | 0.35 | 0.33 | 0.63 | 0.49 | 0.59 |
| 0.5 | 1 | 0.62 | 0.58 | 0.56 | 0.54 | 0.77 | 0.75 | 0.62 | 0.61 |
| 0.5 | 0.75 | 0.56 | 0.50 | 0.50 | 0.47 | 0.76 | 0.74 | 0.65 | 0.63 |
| 0.5 | 0.5 | 0.52 | 0.45 | 0.47 | 0.44 | 0.76 | 0.73 | 0.66 | 0.64 |
| 0.5 | 0.25 | 0.49 | 0.42 | 0.44 | 0.41 | 0.73 | 0.72 | 0.65 | 0.63 |
| 0.5 | 0.15 | 0.47 | 0.41 | 0.43 | 0.40 | 0.72 | 0.71 | 0.64 | 0.63 |
| 0.5 | 0.1 | 0.45 | 0.40 | 0.42 | 0.40 | 0.73 | 0.70 | 0.63 | 0.62 |
| 0.5 | 0.05 | 0.43 | 0.39 | 0.40 | 0.39 | 0.71 | 0.69 | 0.61 | 0.61 |
| 0.5 | 0 | 0.30 | 0.38 | 0.28 | 0.36 | 0.16 | 0.65 | 0.50 | 0.59 |
| 0.25 | 1 | 0.59 | 0.56 | 0.54 | 0.53 | 0.75 | 0.73 | 0.60 | 0.59 |
| 0.25 | 0.75 | 0.56 | 0.51 | 0.50 | 0.48 | 0.75 | 0.73 | 0.63 | 0.61 |
| 0.25 | 0.5 | 0.53 | 0.47 | 0.48 | 0.45 | 0.76 | 0.73 | 0.65 | 0.63 |
| 0.25 | 0.25 | 0.51 | 0.45 | 0.46 | 0.43 | 0.76 | 0.73 | 0.65 | 0.63 |
| 0.25 | 0.15 | 0.50 | 0.44 | 0.44 | 0.42 | 0.74 | 0.72 | 0.64 | 0.63 |
| 0.25 | 0.1 | 0.49 | 0.43 | 0.43 | 0.41 | 0.74 | 0.72 | 0.63 | 0.62 |
| 0.25 | 0.05 | 0.47 | 0.42 | 0.41 | 0.40 | 0.72 | 0.71 | 0.60 | 0.61 |
| 0.25 | 0 | 0.26 | 0.41 | 0.23 | 0.34 | 0.04 | 0.66 | 0.45 | 0.58 |
| 0.15 | 1 | 0.56 | 0.55 | 0.52 | 0.52 | 0.74 | 0.72 | 0.58 | 0.58 |
| 0.15 | 0.75 | 0.54 | 0.50 | 0.49 | 0.48 | 0.75 | 0.72 | 0.61 | 0.60 |
| 0.15 | 0.5 | 0.53 | 0.48 | 0.47 | 0.45 | 0.75 | 0.73 | 0.63 | 0.62 |
| 0.15 | 0.25 | 0.51 | 0.45 | 0.46 | 0.43 | 0.75 | 0.73 | 0.64 | 0.63 |
| 0.15 | 0.15 | 0.50 | 0.45 | 0.44 | 0.42 | 0.75 | 0.73 | 0.63 | 0.63 |
| 0.15 | 0.1 | 0.49 | 0.44 | 0.43 | 0.41 | 0.73 | 0.72 | 0.62 | 0.62 |
| 0.15 | 0.05 | 0.47 | 0.43 | 0.40 | 0.40 | 0.72 | 0.71 | 0.59 | 0.61 |
| 0.15 | 0 | 0.23 | 0.41 | 0.14 | 0.29 | 0.04 | 0.63 | 0.35 | 0.54 |
| 0.1 | 1 | 0.54 | 0.54 | 0.51 | 0.51 | 0.73 | 0.71 | 0.57 | 0.57 |
| 0.1 | 0.75 | 0.52 | 0.50 | 0.48 | 0.47 | 0.73 | 0.72 | 0.60 | 0.60 |
| 0.1 | 0.5 | 0.51 | 0.47 | 0.47 | 0.45 | 0.75 | 0.72 | 0.62 | 0.61 |
| 0.1 | 0.25 | 0.50 | 0.46 | 0.45 | 0.43 | 0.71 | 0.73 | 0.63 | 0.62 |
| 0.1 | 0.15 | 0.49 | 0.44 | 0.43 | 0.42 | 0.73 | 0.73 | 0.62 | 0.62 |
| 0.1 | 0.1 | 0.48 | 0.44 | 0.42 | 0.40 | 0.74 | 0.72 | 0.61 | 0.62 |
| 0.1 | 0.05 | 0.46 | 0.43 | 0.39 | 0.39 | 0.69 | 0.71 | 0.58 | 0.60 |
| 0.1 | 0 | 0.18 | 0.41 | 0.11 | 0.24 | 0.02 | 0.58 | 0.23 | 0.51 |
| 0.05 | 1 | 0.50 | 0.53 | 0.48 | 0.50 | 0.71 | 0.70 | 0.55 | 0.56 |
| 0.05 | 0.75 | 0.49 | 0.49 | 0.47 | 0.47 | 0.72 | 0.71 | 0.58 | 0.58 |
| 0.05 | 0.5 | 0.49 | 0.47 | 0.45 | 0.45 | 0.71 | 0.71 | 0.60 | 0.60 |
| 0.05 | 0.25 | 0.48 | 0.45 | 0.43 | 0.42 | 0.72 | 0.72 | 0.61 | 0.61 |
| 0.05 | 0.15 | 0.47 | 0.44 | 0.41 | 0.41 | 0.71 | 0.72 | 0.60 | 0.61 |
| 0.05 | 0.1 | 0.46 | 0.44 | 0.38 | 0.39 | 0.69 | 0.72 | 0.58 | 0.61 |
| 0.05 | 0.05 | 0.44 | 0.43 | 0.35 | 0.37 | 0.67 | 0.71 | 0.52 | 0.59 |
| 0.05 | 0 | 0.11 | 0.40 | 0.03 | 0.17 | 0.02 | 0.43 | 0.05 | 0.36 |
| 0 | 1 | 0.23 | 0.52 | 0.30 | 0.49 | 0.05 | 0.68 | 0.49 | 0.54 |
| 0 | 0.75 | 0.25 | 0.48 | 0.31 | 0.46 | 0.04 | 0.69 | 0.49 | 0.56 |
| 0 | 0.5 | 0.25 | 0.45 | 0.29 | 0.43 | 0.03 | 0.69 | 0.48 | 0.58 |
| 0 | 0.25 | 0.23 | 0.43 | 0.25 | 0.40 | 0.02 | 0.69 | 0.39 | 0.58 |
| 0 | 0.15 | 0.18 | 0.42 | 0.17 | 0.37 | 0.02 | 0.67 | 0.28 | 0.55 |
| 0 | 0.1 | 0.15 | 0.40 | 0.14 | 0.34 | 0.02 | 0.66 | 0.18 | 0.54 |
| 0 | 0.05 | 0.07 | 0.36 | 0.04 | 0.27 | 0.01 | 0.60 | 0.07 | 0.46 |
