# Supplementary material for: Phenomic and genomic prediction of yield on multiple locations in winter wheat
Source: Front Genet. 2023 May 9;14:1164935. doi: 10.3389/fgene.2023.1164935 (PMC10203586; doi:10.3389/fgene.2023.1164935)
Supplement: Supplementary file 2 [file Table2.DOCX]

**Supplemental Table 1:** Variables measured during the trials in seasons 2015/2016 and 2016/2017 at two locations in each season. For harvest, field scoring and soil measurements, the (starting) date of the measurement is given. For the remote sensing data (multispectral and hyperspectral) the dates of measurement are stated on top, and the collected traits are indicated by “x”. Further information on individual vegetation indices can be found in Odilbekov et al. (2018) and references therein, and the IndexDatabase (<https://www.indexdatabase.de/>).

[Odilbekov, F., Armoniene, R., Henriksson, T., & Chawade, A. (2018). Proximal Phenotyping and Machine Learning Methods to Identify Septoria Tritici Blotch Disease Symptoms in Wheat. *Frontiers in Plant Science*, *9*, 685. <https://doi.org/10.3389/fpls.2018.00685> ]

| **Source** | **Trait** | **Abbreviation** | **Measurement date (s)** | |  |  |
| --- | --- | --- | --- | --- | --- | --- |
|  |  |  | **Cambridge 2016** | **Duxford 2016** | **Duxford 20167** | **Hinxton 2017** |
| **Drilling** |  |  | 23/10/2015 | 26/10/2015 | 12/10/2016 | 24/10/2016 |
|  |  |  |  |  |  |  |
| **Harvest** | Raw weight | RW | 03/09/2016 | 31/08/2016 | 15/08/2017 | 25/08/2017 |
|  | Moisture contents | MC | 03/09/2016 | 31/08/2016 | 15/08/2017 | 25/08/2017 |
|  | Hectoliter weight | HLW |  |  | 15/08/2017 | 25/08/2017 |
|  | Yield | YLD | 03/09/2016 | 31/08/2016 | 15/08/2017 | 25/08/2017 |
| **Field walk** | Establishment | EST |  |  | 16/11/2016 | 23/11/2016 |
|  | Initial crop cover | ICC |  |  | 16/11/2016 | 23/11/2016 |
|  | green | GRN | 19/07/2016 | 19/07/2016 |  |  |
|  | wax | WAX | 12/07/2016 | 12/07/2016 | 15/06/2017 | 21/06/2017 |
|  | wax segregation | WSG | 12/07/2016 | 12/07/2016 | 15/06/2017 | 21/06/2017 |
|  | YR | YR | 28/04/2016 | 05/04/2016 |  |  |
|  | Growth stage EARLY | GSE | 26/05/2016 | 26/05/2016 | 24/05/2017 | 26/05/2017 |
|  | Growth stage MID | GSM | 06/06/2016 | 06/06/2016 | 31/05/2017 | 02/06/2017 |
|  | Growth stage LATE | GSL | 13/06/2016 | 13/06/2016 | 07/06/2017 | 09/06/2017 |
|  | Necrosis | NEC |  |  | 26/04/2017 | 24/04/2016 |
|  | Height | HGT |  |  | 03/07/2017 | 07/07/2017 |
|  | Height variation | GHV |  |  | 03/07/2017 | 07/07/2017 |
|  | Awns | AWN |  |  | 13/06/2017 | 14/06/2017 |
|  | Diseased | DIS |  |  | 13/06/2017 | 14/06/2017 |
| **Soil measurement** | Conductivity deep | SCD | 25/04/2016 | 25/04/2016 | 02/05/2017 | 02/05/2017 |
|  | Conductivity shallow | SCS | 25/04/2016 | 25/04/2016 | 02/05/2017 | 02/05/2017 |
|  |  |  |  |  |  |  |
| **Multispectral** |  |  | 19/04/2016 | 19/04/2016 | 06/04/2017 | 07/04/2017 |
|  |  |  | 06/06/2016 | 06/06/2016 | 10/05/2017 | 10/05/2017 |
|  |  |  | 21/07/2016 | 21/07/2016 | 03/06/2017 | 03/06/2017 |
|  |  |  | 09/08/2016 | 09/08/2016 | 27/06/2017 | 04/07/2017 |
|  |  |  |  |  | 12/07/2017 | 12/07/2017 |
|  | Digital Surface Model | dsm | x | x | x | x |
|  | Digital Terrain Model | dtm | x | x | x | x |
|  | Enhanced Normalized Difference Vegetation Index | ENDVI | x | x |  |  |
|  | Generalized Difference Vegetation Index | GDVI | x | x | x | x |
|  | Green leaf Index | GLI | x | x |  |  |
|  | Green Normalized Difference Vegetation Index | GNDVI | x | x |  |  |
|  | Green Soil Adjusted Vegetation Index | GSAVI | x | x |  |  |
|  | Height max | HGmax |  |  | x | x |
|  | Height mean | HGmean |  |  | x | x |
|  | Height min | HGmin |  |  | x | x |
|  | Normalized Difference Green/Red Normalized green red difference index | NGRDI | x | x |  |  |
|  | Normalized Difference Red Edge Index | NDRE |  |  | x | x |
|  | Normalized Difference Vegetation Index | NDVI |  |  | x | x |
|  | Optimized Soil Adjusted Vegetation Index | OSAVI |  |  | x | x |
|  | Visible Atmospherically Resistant Index | VARI | x | x |  |  |
| **Hyperspectral** |  |  | 27/06/2016 | 27/06/2016 | 07/07/2017 | 07/07/2017 |
|  | Anthocyanin Reflectance Index 1 | ARI1 | x | x | x | x |
|  | Anthocyanin Reflectance Index 2 | ARI2 | x | x | x | x |
|  | Anthocynin Content Index | ACI | x | x | x | x |
|  | Canopy height | CH |  |  | x | x |
|  | Carotenoid Reflectance Index 1 | CRI1 | x | x | x | x |
|  | Carotenoid Reflectance Index 2 | CRI2 | x | x | x | x |
|  | Carotenoid Reflectance Index Green | CRIG | x | x | x | x |
|  | Carotenoid Reflectance Index Red Edge | CRIRE | x | x | x | x |
|  | Cellulose Absorption Index | CAI | x | x | x | x |
|  | Chlorophyll Absorption Ratio Index | CARI | x | x | x | x |
|  | Chlorophyll Index Green | CIG | x | x | x | x |
|  | Chlorophyll Index Red Edge | CIRE | x | x | x | x |
|  | Clay Absorption Depth | CAD | x | x | x | x |
|  | Enhanced Vegetation Index | EVI | x | x | x | x |
|  | Forest Area Boost Index | FABI | x | x | x | x |
|  | Hematite Index (RI) | Hm | x | x | x | x |
|  | Hydrocarbon Index | HI | x | x | x | x |
|  | Inverted Red Edge Chorophyll Index | IRECI | x | x | x | x |
|  | Iron Absorption Depth 1 | IAD1 |  |  | x | x |
|  | Iron Absorption Depth 2 | IAD2 |  |  | x | x |
|  | Leaf Chlorophyll Index | LCI | x | x | x | x |
|  | Lidar_dtm | ldtm |  |  | x | x |
|  | Modified Anthocyanin Reflectance Index | MARI | x | x | x | x |
|  | Modified Chlorophyll Absorption Ratio Index | MCARI1 | x | x | x | x |
|  | Modified Chlorophyll Absorption Ratio Index 2 | MCARI2 | x | x | x | x |
|  | Modified Normalized Difference 680 | MND680 | x | x | x | x |
|  | Modified Normalized Difference 705 | MND705 | x | x | x | x |
|  | Modified Normalized Difference Built-up Index | MNDBI | x | x | x | x |
|  | Modified Red Edge NDVI | MNDVI705 | x | x | x | x |
|  | Modified Simple Ratio | MSR | x | x | x | x |
|  | Modified Soil Adjusted Vegetation Index 2 | MSAVI2 | x | x | x | x |
|  | Moisture Stress Index | MSI | x | x | x | x |
|  | Normalized Difference Built-up Index | NDBI | x | x | x | x |
|  | Normalized Difference Infrared Index | NDII | x | x | x | x |
|  | Normalized Difference Lignin Index | NDLI | x | x | x | x |
|  | Normalized Difference Nitrogen Index | NDNI | x | x | x | x |
|  | Normalized Difference Vegetation Index | NDVI | x | x | x | x |
|  | Normalized Difference Water Index | NDWI | x | x | x | x |
|  | Normalized Multiband Drought Index | NMDI | x | x | x | x |
|  | Normalized Pigment Chlorophlyll Index | NPCI | x | x | x | x |
|  | Normalized Soil Moisture Index | NSMI | x | x | x | x |
|  | Optimized Soil Adjusted Vegetation Index | OSAVI | x | x | x | x |
|  | Photochemical Reflectance Index | PRI | x | x | x | x |
|  | Pigment Index 2 | PI2 | x | x | x | x |
|  | Pigment Specific Normalized Difference | PSND | x | x | x | x |
|  | Pigment Specific Normalized Difference 2 | PSND2 | x | x | x | x |
|  | Pigment Specific Simple Ratio | PSSR | x | x | x | x |
|  | Plant Biochemical Index | PBI | x | x | x | x |
|  | Plant Senescence Reflectance Index | PSRI | x | x | x | x |
|  | Plant Water Band Index | PWBI | x | x | x |  |
|  | Ratio Vegetation Index | RVI | x | x | x | x |
|  | Red Edge Area 1 | REA1 |  |  | x | x |
|  | Red Edge Area 2 | REA2 |  |  | x | x |
|  | Red Edge Area 3 | REA3 |  |  | x | x |
|  | Red Edge Normalized Difference Vegetation Index | RENDVI | x | x | x | x |
|  | Red Edge Normalized Difference Vegetation Index 2 | RENDVI2 | x | x | x |  |
|  | Red Edge Position | REP | x | x | x | x |
|  | Red Edge Position Index (linear extrapolation) | REPI-ex |  |  | x | x |
|  | Red Edge Position Index (linear interpolation) | REPI-in | x | x | x | x |
|  | Red Edge Vegetation Stress Index | REVSI | x | x | x | x |
|  | Red Green Ratio Image | RGRI | x | x | x | x |
|  | Salinity Index | SI | x | x | x | x |
|  | Simple Index | DVI | x | x | x | x |
|  | Soil Adjusted Vegetation Index | SAVI | x | x | x | x |
|  | Soil Organic Carbon 1 | SOC1 | x | x | x | x |
|  | Soil Organic Carbon 2 | SOC2 | x | x | x | x |
|  | Soil Organic Carbon 3 | SOC3 | x | x | x | x |
|  | Structure Insensitive Pigment Index | SIPI |  |  | x | x |
|  | SWIR Fine particles Index | SWIR |  |  | x | x |
|  | Vegetation Index Green | VIG | x | x | x | x |
|  | Visible Atmospherically Resistant Index | VARI | x | x | x | x |
|  | Vogelmann Red Edge 3 | VRE3 | x | x | x | x |
|  | Water Band Index | WBI | x | x | x | x |
|  | WorldView Built-up Index | WBuI | x | x | x |  |
|  | WorldView Water Index | WWI |  |  | x | x |
|  |  |  |  | ````````````````````````` |  |  |
|  |  |  |  |  |  |  |
